# Supplementary material for: Splice-Junction-Based Mapping of Alternative Isoforms in the Human Proteome
Source: Cell Rep. Author manuscript; Available in PMC 2020 Jan 15. (PMC6961840; doi:10.1016/j.celrep.2019.11.026)
Supplement: 2 [file NIHMS1546469-supplement-2.zip › DF1/PXD010154/Thyroid/Thyroid_29_DNAJC27_LTSLIWLDIPSSMREPGRSLR.pdf]

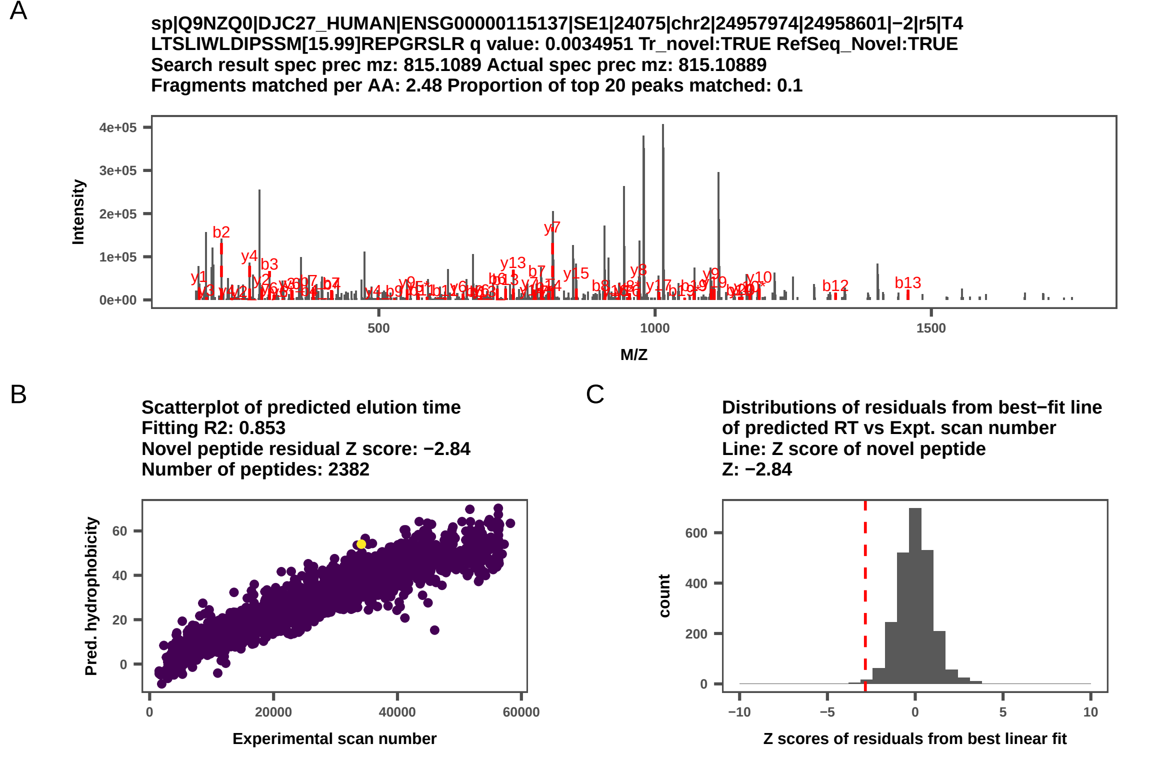
A
sp|Q9NZQ0|DJC27_HUMAN|ENSG00000115137|SE1|24075|chr2|24957974|24958601|−2|r5|T4
LTSLIWLDIPSSM[15.99]REPGRSLR q value: 0.0034951 Tr_novel:TRUE RefSeq_Novel:TRUE
Search result spec prec mz: 815.1089 Actual spec prec mz: 815.10889
Fragments matched per AA: 2.48 Proportion of top 20 peaks matched: 0.1
Intensity
4e+05
3e+05
2e+05
1e+05
0e+00
b2
y4
b3
y1
y7
y13
b7
y15
y8
y9
y10
b12
b13
500
1000
1500
M/Z
B
Scatterplot of predicted elution time
Fitting R2: 0.853
Novel peptide residual Z score: −2.84
Number of peptides: 2382
Pred. hydrophobicity
60
40
20
0
0
20000
40000
60000
Experimental scan number
C
Distributions of residuals from best−fit line
of predicted RT vs Expt. scan number
Line: Z score of novel peptide
Z: −2.84
count
600
400
200
0
−10
−5
0
5
10
Z scores of residuals from best linear fit
